# Minding the gap between artificial and biological computing paradigms for biologically loyal AI

**DOI:** 10.3389/fnsys.2025.1695493

**Published:** 2026-01-13

**Authors:** K. L. Kirkpatrick

**Affiliations:** Departments of Mathematics and Physics, University of Illinois Urbana-Champaign, Urbana, IL, United States

**Keywords:** biological computing, biologically loyal AI, cognition, computability, multiscale neurobiology, neural networks, proof theory, whole brain emulation

## Abstract

The theoretical foundation of neuroscience differs from that of artificial intelligence, and to bridge this gap with AI, we would need a new computing paradigm that describes both fields well. The gap came from mathematicians’ invention of computability theory, which was deliberately narrower than cognition and yet became a cornerstone of computer science and cognitive science. It has resulted in circular logics for computational biology and biological computing: the computability model of human mathematical activities can limit the sort of technology we build, and in turn, the engineering constraints on our technologies can limit our understanding of brain systems. Here we study several important mathematical and biological activities that computability neglects, helping to bridge the gap between neurobiology and (aspirational) AGI. One such activity is mathematicians’ producing proofs of theorems that lie outside artificial computers’ logic. Another is neurons’ functions that are more complex than transistors, informed by recent neurobiological findings. We end by surveying candidates and inspiration for a new synthesis of AGI with neurobiology, presenting the hypothesis that a new paradigm would have to thoroughly integrate cognition and motion.

## 1 Introduction

Technology companies have put forward artificial intelligence (AI) models as if the models are autonomous. They are not. A secret of the AI world is that there are legions of humans supporting each AI model, and yet the models often get worse over time, as in the examples of ChatGPT (Chen et al., 2024; Shumailov et al., 2024). Additionally, AI models need vast amounts of human help for preparing data, training through feedback, checking output, and correcting mistakes (Kantrowitz, 2023).

The term AI originated in the 1950s within academia to replace “computer simulation” and is misleading: AI uses a lot of non-artificial resources, especially human efforts, and it’s not very “intelligent” (an objectionable term on its own). The term AGI (artificial general intelligence) originated more recently within industry and has similar problems. Both terms will be used in this article, with an awareness of their issues. Some history of the term “computer” may also be helpful. From the early 1600s to the early 1940s, the word “computer” meant a human mathematician who did computations, often with the aid of calculating machines such as slide rules. Around 1945, “computer” came to mean a general-purpose electronic machine, able to execute a variety of programs (OED, 2025).

Why does so-called AI need so much help from humans? The answer may lie in the theory that underpins computer science: there is a gap between artificial and biological computing. This means that any AI or AGI based on current computing theory will lack the functions in the gap; for some sort of true AI or AGI, we would need a new computing theory that bridges this gap.

Without awareness of the gap between devices and biology, there is a circular logic behind AI, similar to the logic behind the concept of a genetic program (Keller, 2000). For AI, mathematicians and scientists took human ingenuity, reduced it to only the only key parts (or so they thought), and called it computability theory. Then engineers built technologies that mimic only those key parts (called computers and computer science), and some engineers claimed to have replicated (parts of) human ingenuity (in aspirational AI or AGI). But AI has failed to “live” up to the hype, so perhaps the parts of cognition that were left out from computation may turn out to be important also. We may want to add some of those things back in, if we want devices that are more like us.

A similar circular logic may happen in parts of computational neuroscience unless there’s awareness of these issues. If scientists try to explain neurobiological systems by computational models alone, there will be important things left out. But good modeling in neuroscience has awareness of these issues, considering bodies and peripheral structures of the nervous systems to be important to the neural model, as well as geometry in the environment (Golowasch et al., 2002; O’Leary et al., 2015; Norekian et al., 2024; Curto and Sanderson, 2025; and others).

The article is arranged in sections as follows: (2) We discuss the historical divergence by mathematicians from broad cognition to narrow computability, of which there are many equivalent formulations and on which current AI models are founded. The broader aspects of cognition left out of computability are crucial for reasoning and learning, and are unlikely for current AI models to achieve. (3) We motivate the examination of gaps between humans and machines that a new synthesis of AGI and neuroscience must consider and bridge. (4) We provide a new analysis of certain theorem-proving abilities of human ingenuity and there being no evident way to map these human abilities onto machine design, because of the digital logic and design of current machines. This is a macroscopic gap between human computers and artificial computers. (5) We also interpret recent neurobiological findings in light of the mathematical assumptions that underpin current AI models. These findings illuminate gaps between the microscopic parts of brains (especially sub-neuronal structures) and the microscopic parts of machines (especially microchip structures). These microscopic gaps may be at the root of the macroscopic differences that we observe, and they should shed light on neural mechanisms of human ingenuity. (6) We examine the multiscale nature of brains, which seems to increase efficiency and ingenuity, and which should provide lessons for making better devices.

Finally, in the discussion section (7), we survey inspirations for a new synthesis of AGI with neurobiology, both theoretical and technological. A specific hypothesis, that cognition is fundamentally entangled with motion at all scales, is proposed in detail there too.

## 2 Mathematicians have historically pivoted from cognition to computability

Of the many functions of cognition, the macroscopic activities of mathematicians that we will focus on are calculating things and proving theorems. These activities involve other cognitive skills, from sophisticated skills such as problem-solving, through learning, understanding, and explaining, to fundamental skills such as attention, perception, and memory. It is often said during proof-based mathematics courses that one cannot truly understand a fact until one has proved it. This is because many proofs (the constructive ones, at least) also explain why a fact is true. When we construct a proof for the first time, we are putting together previously known facts into a flow of logic. And mathematicians don’t need massive data sets like LLMs do; for most proofs, we can learn from a few examples together with underlying principles.

But computer scientists may not be able to fully systematize the activity of proving theorems, due to incompleteness theorems in logic. Proving theorems can also be a complicated and messy process, perhaps too challenging to pin down. Instead, mathematicians have historically systematized the activity of calculating or computing from algorithms, a subfield called computability theory. Computability is narrower than proving theorems and much narrower than cognition; but computability is more concrete and has had success both as a theory and as a basis for technologies.

This results in an asymmetry between people and artificial computers: humans can emulate the computational activities of artificial computers, but not vice versa, as expanded later on in the discussion around completeness and incompleteness theorems by Gödel, Turing, and Feferman. The upshot is that electronic computers based on existing hardware and theory may not be able to achieve the same logical sophistication as logicians.

### 2.1 Turing’s version of computability was the a-machine

To simulate the calculating activities of a mathematician with pen and paper, Alan Turing in 1936 defined the automatic a-machine (now called a Turing Machine or TM) (Turing, 1936). These activities include reading a symbol on a tape, erasing a symbol, writing a symbol, moving the tape forward or back, changing the internal state of the machine, and following the next operation-and finite combinations of these activities.

The machine itself is abstract and has several abstract parts: A tape with an unbounded number of squares, a head that does the reading and writing of each square, and a state register, together with a finite set of symbols (including the blank space as a symbol), a finite set of states (including a final Halt state), and a finite instruction table that determines the next operation from the state and the symbol in the square under the head. Artificial computers are often thought of as instantiating TMs, which is approximately true, because memory registers are bounded, unlike a TM tape.

### 2.2 Mathematicians have many versions of computability, and they are equivalent

Turing’s machines may be the most famous model of computing, but they weren’t the first, and they are equivalent to other models that have been proposed. Before computability, there was recursion theory, founded and developed in the early 1930s by Rózsa Péter and others (e.g., Péter, 1935). Recursion theory came to be supplanted in name by computability theory; the two theories are equivalent (Fernández, 2009). To be specific, the set of functions that can be computed by TMs are exactly the set of *general recursive functions*, each a map from the set of natural numbers to itself, a map that is formally and intuitively computable.

In the later 1930s, Alonzo Church formulated the lambda calculus, which is a way of manipulating logical expressions using substitution, function evaluation, and other operations called reductions—kind of like algebra rules for logic (Church, 1936b,1936a). (Church’s earlier attempt in 1932 was shown to be inconsistent by Kleene and Rosser in 1935.) The lambda calculus turns out to be logically equivalent to Turing machines and to general recursive functions (Turing, 1937). In other words, the lambda calculus is Turing complete.

Over the years, other models of computing have been proposed and developed; some have been shown to be Turing complete, and others, less capable than TMs. Some of the models less capable than TMs include finite state automata and pushdown machines. Some of the Turing-complete models include: Post-Turing machines in the late 1930s (Post, 1927–1991), cellular automata in the late 1940s (von Neumann, 1951), and Markov algorithms around 1960 (Markov, 1960). Some models were designed to describe concurrent or parallel computing, and thought to be more sophisticated than Turing Machines, because intuitively it seems that parallel computing is more powerful than traditional sequential computing. These models include actor models and process calculi in the 1970s (Greif, 1975; Baeten, 2004). But these also were proved to be equivalent to TMs, which was something of a surprise (but maybe should not have been, in light of other equivalences in mathematics, such as the fact that the set of integers is the same size as the set of rational numbers).

Other variations on TMs may sound more powerful than TMs on the surface, but they also turn out to be computationally equivalent to TMs. These include non-deterministic TMs, multi-tape TMs, multi-head TMs, and multi-dimensional TMs (where the head of the machine can move in a 2-dimensional grid).

### 2.3 Efforts to get beyond Turing computability are either useful or realizable, but not both

Turing described an oracle that could compute a non-recursive function and thus be more powerful than a TM; this cannot be built, however. These kinds of models of computing better than Turing complete are called hypercomputers. Other hypercomputers have been proposed, such as machines inspired by Zeno’s paradox, random or fuzzy-logic-based TMs, standard computers moving at light-speed, real-valued computers such as Blum–Shub–Smale (BSS) machines, and non-standard quantum computers if they can handle infinitely many states.

These models of computing have drawbacks. Some are purely abstract and cannot be implemented in reality, such as BSS machines (Siegelmann, 1998; Aaronson, 2005). Others can be made but are of limited use, such as random TMs, since all they do is generate random quantities.

### 2.4 McCulloch and Pitts claimed that neural computing explains cognition

The computational theory of cognition and the architectures of artificial neural networks (ANNs) have roots in the early 1940s (McCulloch and Pitts, 1943). McCulloch and Pitts used Turing’s computability framework to construct a model of neural computing, with nets of nodes they likened to neurons. They made a number of assumptions in order to boil brain activity down to (binary digital) logic, and some of these assumptions have been propagated into the ANNs underlying LLMs (large language models) and other current AI models. These assumptions may reveal gaps, however, between the hopes of ANNs and the realities of neurons in brains, partly because since the 1940s neuroscience has made more progress in understanding many details of brain cell functions than computer science has made in adding details to artificial “neuron” functions.

For example, McCulloch and Pitts assumed that the “nervous system is a net of neurons,” and in the 1940s, this may have seemed true, because little was known about the nerve cells that are not neurons, called glia (The name “glia,” meaning “glue,” shows how little credit these cells got). In reality, glial cells are crucial in the nervous system and started being studied earnestly in the second half of the 20th century. We now know that some glia are excitable much like neurons, and that some glia are crucial in learning through their influence on synapses. Thus it would be more accurate to assume that the nervous system is a conglomeration of neurons and glia, partly in networks and partly in non-network structures (such as the microglia that move around freely among other nerve cells).

McCulloch and Pitts also assumed that the neuron’s activity is all or none, i.e., that the only significant neural event is a binary, excited impulse (a.k.a. spike) sent from the neuron’s center (a.k.a. soma) along an axon to the end and then passing a signal across a synapse to another neuron’s dendrite (the branch of a tree-like structure considered “upstream” from the trunk-like soma). But we now know that dendrites can also send spikes up the dendritic tree away from the soma, called back-propagating spikes (not the same as the backpropagation algorithm for training ANNs) (Short et al., 2017). Moreover, it used to be assumed that only a spike down the axon can cause the release of neurotransmitters. However, dendrites themselves release neurotransmitters (Ludwig et al., 2016), and the soma also does (Welle et al., 2018). Thus it would be more accurate to include a broader range of activities among important nervous functions, both binary and non-binary, both excited and mellow.

They also assumed that a fixed number of (dendritic) synapses must be stimulated in order to produce a spike, explicitly assuming this is independent of prior activity (it’s not, due to plasticity) and of location in the dendritic arbor. But there is no such independence, because dendritic shape can be complicated and can result in sophisticated signaling (Li et al., 2015). And different orders of signals arriving at dendrites can have different outcomes (Feldman, 2012; Nevian and Sakmann, 2004; Rall, 1964). McCulloch and Pitts also assumed other things that should be re-evaluated: that any inhibition is absolute and completely silences the neuron, and that the net’s structure is static in time. More fundamentally, they made claims, summarized cogently, “that neural activity is computation and that neural computation explains cognition” (Piccinini and Bahar, 2013). These claims of McCulloch and Pitts are probably false based on what we know now about nerve cells and based on what we will discuss about cognition in the section on theorem-proving as an important cognitive function. In particular, the McCulloch and Pitts idea that neural computation is digital (and became known as the computational theory of cognition) has been persuasively refuted in favor of a uniquely neural kind of computation that is also not analog (Piccinini and Bahar, 2013).

The McCulloch and Pitts model influenced later artificial neural networks, such as the perceptrons of the mid-20th century (Rosenblatt, 1958) and deep artificial neural networks of the early 21st century (Schmidhuber, 2015). Some of McCulloch and Pitts’s assumptions have been changed in deep ANNs, such as replacing binary classifiers by other activation functions like sigmoidal curves (summarized in, e.g., Krauss, 2023). Most current AI models have inherited many of McCulloch and Pitts’s assumptions, such as excluding glial cells and excluding dendrites’ activities beyond summing, such as their reconfiguring their geometry, analyzing the order of inputs, and releasing gases or neurotransmitters. On the other hand, there are carefully constructed models that are more biologically realistic, such as dendritic neuron models (DNMs) (Wang and Liu, 2025) and spiking neural networks (SNNs) (Tavanaei et al., 2018).

These exclusions implicate cognition and illustrate why we should attend to differences at both microscopic and macroscopic levels between real brains and artificial networks. New versions of brain network models would need to share more important features with real brain cells (microscopic), real brain networks (mesoscopic), real organisms (macroscopic), and their social and natural environments (megascopic) in order to get closer to a synthesis of neurobiology and AGI. But as long as AI models are built with oversimplified “neurons” on digital computing devices, they cannot capture the human reasoning that requires logic beyond what digital logics can reach. (This latter point will become more precise soon, in section 4).

## 3 New paradigms must address gaps between human computers and artificial computers

There are gaps between the abilities of artificial computers and of human computers, on various space-time scales and in various domains. These gaps span the scales from nanometers and femtoseconds for small molecules up through microns and seconds to meters and months for humans. And these gaps span domains from actions and decisions to calculations and proofs, and various combinations of these.

There are convergences between artificial and human computers, of course. But we must describe the gaps well enough for theorists to formulate a new synthesis of fundamental neurobiology and aspirational artificial general intelligence (AGI), and for empiricists to understand the evolution and neuronal mechanisms of cognitive abilities, and then to get similar abilities from technology with similarly low demands for energy and material. This section discusses some of these gaps based on our current knowledge of neurobiology and electronics. More gaps—and more convergences—will likely appear when neuroscientists make new progress and when device users figure out new hacks.

One reason we need a new synthesis, a unifying paradigm for biology and technology, is that we will keep using old technology under a new paradigm, perhaps because it still works or uses less energy. For example, we keep using traditional digital calculators even if LLMs are available, because using ChatGPT as a calculator is inefficient and gives a lot of wrong answers, e.g., getting about 45% of 3-digit multiplication tasks wrong (Dziri et al., 2023). And trying to force ChatGPT to do calculations correctly, through prompt engineering, is more inefficient and still risks errors. Also, most current LLMs run on conventional, if parallel, microchips, so LLMs have no hypothetical edge over traditional calculators at multiplication tasks. There are new devices that implement the architecture of certain ANNs in other types of hardware, such as memristive devices, that have relatively deterministic and static structures and remaining challenges that may result in lower precision (Aguirre et al., 2024). In any case, we will always want to integrate old and new technologies and biology all together into useful systems, so it would help if their theories are all integrated into one unified theory. Ideally, a unifying theory of bio-computing would also cover as-yet-undreamed-of technology, including the kind that may lead to actual AGI.

Pinpointing gaps between artificial and biological computers is needed for a new synthesis of neurobiology with AGI. Without knowing about these gaps, it is hard to explain the many failures of AI models on a wide variety of tasks. These failures of AI include adversarial examples known since the mid-2010s: researchers made a small change to a picture of a panda, and the ANN classified it a “gibbon” even though the picture still looks like a panda to people (Goodfellow et al., 2014). More recently, failures of LLMs include situations where they make up output that is nonsensical, called “hallucinations”—a term that carries anti-disability sentiment and would be better replaced by “non-sense” or “made-up.”

Such failures may seem random, but some of them can be explained. For example, when users asked Google how to keep toppings on a pizza, AI Overviews wrote to use “non-toxic” glue, a piece of advice that turned out to have come from a 2013 sarcastic comment in Reddit (McMahon and Kleinman, 2024; Kelly, 2024).

Explaining these failures is rather piecemeal so far, but if there is a unifying theory for these failures, that would help this field. When organizations try to fix their models’ failures, they may try re-training or prompt engineering first, but because that is inefficient or ineffective, often the LLMs must be scrapped or trained from the start again. LLMs seem to be bad at learning new things on top of what they’ve already “learned:” for instance, adding new data to LLMs can cause nonsensical output or mistaken reasoning later (Gekhman et al., 2024; Huang et al., 2025).

In any case, these failures show that there are important differences between how models “learn” and how people learn. Later, when we discuss microscopic differences, e.g., between neurons and LLM nodes, that will also shed light on how our brain’s abilities expanded over the generations of evolution without demanding overly many neurons or overly much energy. First, however, we look into more important differences at the macroscopic level, between artificial computers and mathematicians in the role of human computers.

## 4 Macroscopic: gaps between mathematicians and artificial computers based on proof theory

In the context of human cognition, two important activities of mathematicians are proving theorems and doing calculations. We start with calculations, with the goal to understand gaps between human computers (mathematicians doing calculations) and artificial computers (electronic devices carrying out programmed instructions). The term “computer” shifted its meaning from humans to devices after the development of recursion theory around the 1930s (Péter, 1935), later known as computability theory (Gödel, 1931; Turing, 1936).

Recursion and computability were meant to capture certain activities of human mathematicians, but not all activities. As before, Turing defined his automatic a-machines to simulate the calculating activities of a mathematician with pen and paper, specifically: reading a symbol on a tape, erasing a symbol, writing a symbol, moving the tape forward or back, changing the internal state of the machine, and following the next operation. But Turing’s framework for computability (and indeed every other framework for computability) does not exhaust all the activities of mathematicians, who can do many more actions, including a number of actions that evidently cannot be reduced to TM actions.

As an aside, mathematicians can do non-mathematical actions, such as physically moving themselves and moving things other than pen and paper, all of which are “definite physical effects” in Turing’s terminology from his National Physical Laboratory report (Turing, 1948). In other words, humans are closely integrated with our “actuators,” such as arms and legs, in contrast to robotics where actuators are separate from central processing units (CPUs).

More to the point, mathematicians can do mathematical actions that cannot be reduced to TM actions. For example, mathematicians can create new methods and algorithms, an activity which is at least partly beyond the reach of any computing device. Certainly, mathematicians can notice mistakes in algorithms or solutions, e.g., by checking with a different method or estimating Fermi-style, and learn from those errors, something that computing systems struggle with (Rahgouy et al., 2023) (finding that LLMs have a long way to go on Fermi reasoning at the human level).

Similarly, programmers can debug software and not get stuck in infinite loops, unlike software. To be sure, there is software that helps with debugging, but it requires human oversight and human actions outside of itself. And any software is limited by the fact that the halting problem is undecidable: there is no computer program that could be written to correctly decide whether any other program and its input will halt or get stuck in an infinite loop. Humans, on the other hand, can decide more reliably whether a program will halt and fix bugs that prevent halting, however frustrating the debugging process may be. To be fair, there may be software bugs that humans cannot find, although hackers’ endless exploits are evidence that people can find bugs if they’re motivated enough.

On the other hand, there are situations where programmers should not even try to find and fix all software bugs. Good examples are the Mars landers, which each had millions of lines of code. It was impractical to debug them completely, partly because each debugging fix has a small probability of introducing a new bug. For NASA, it was more practical to do workarounds for each of the known bugs. And mathematicians at NASA developed a statistical model of bugs in the code, both for how the bugs originate and how they manifest in operations (Taber and Port, 2014). This model helped NASA determine how reliable the landers were, with precision so high that the landings were successful and became more successful.

This human ingenuity, using and making sophisticated tools, is what has accomplished fantastic things on Mars. We may be tempted to view space robots as fully autonomous, but their existence and functioning depend on people (similar to LLMs, in fact). Nevertheless, robots like the Mars ones are more likely to achieve human-like abilities than LLMs, because they are required to do and are designed to do physical actions other than information processing—similar to mathematicians. Surely, the necessity of combining actions and computations has been a main cause of human ingenuity, and it could be a cause of artificial ingenuity as well. But we must avoid the temptation to simply tack on “action” to AI models, and we must instead think hard about what robots need to do and how to do those things well (which may or may not involve LLMs or the like).

### 4.1 Mathematicians can prove theorems that artificial computers can never prove

Human mathematicians can do another thing that is not reducible to Turing Machine actions: proving theorems that are unprovable in the logical framework of TMs that underpins artificial computers. To be fair, artificial computers can prove a number of theorems, and they have been helpful for assisting proofs of some challenging theorems, such as the Four Color Theorem. But there are theorems that artificial computers can state but cannot prove—and this is a provable fact.

Proving that something is unprovable sounds like a weird thing that perhaps only mathematicians or lawyers would do, but it is an example of Gödel’s incompleteness theorems. Gödel proved, among other things, that if we construct an algorithm that can list theorems based on a given set of axioms consistently, then there are true things that the algorithm can state but cannot prove or disprove (Gödel, 1931; see also Feferman, 1986). In any given formal system, there are some theorems that are true but every algorithm in that axiomatic system would fail at proving.

Thus, artificial computers cannot construct proofs of theorems that are unprovable in the device logic, no matter how well they are programmed and no matter much power and scale they have. I am using the word “construct” here to mean “create genuinely,” that is, create by means other than, for example, copying or random luck. Mathematicians, on the other hand, can prove some of these theorems that artificial computers cannot. The reason is that we can create other logic systems beyond those of artificial computers, and we can construct proofs in these other logic systems that artificial computers cannot reach.

There is an asymmetry between human and artificial computers: humans can emulate the mathematical activities of artificial computers, but not vice versa. Devices can imitate our outputs and can simulate our calculations, but they cannot reproduce all of our related processes.

Interestingly, Turing believed the opposite, that there is no abstract obstacle to building computers as logically powerful as humans, contrary to the “mathematical objection to machine intelligence” (Piccinini, 2003). He proved a theorem in his 1939 dissertation that found a narrow circumstance not covered by Gödel’s incompleteness theorems, and then he made a conjecture there that this kind of partial completeness result could be pushed further to overcome the mathematical objection (Appel, 2012). This conjecture turned out to be false, as shown in a 1962 proof (Feferman, 1962). As a result it is still a viable hypothesis that electronic computers based on existing hardware and theory cannot achieve the same logical sophistication as logicians. An updated mathematical objection to machine intelligence is still live.

To be specific, Turing tried to prove in 1939 that an infinite sequences of axiomatic systems, called an “ordinal logic” in the limit, be used to prove any theorem in a class of mathematical statements. He wanted this to be true for a sweeping class of mathematical statement, but he was able to prove it only for the class of statements of the form “for all x, *f*(x) = 0,” where f is primitive recursive, a class now called $\Pi_{1}^{0}$. He conjectured that this same ordinal logic would be complete for the larger class of “number-theoretic statements” in his terminology, now called $\Pi_{2}^{0}$. This larger class of statements is of interest because some arithmetical and number-theoretic statements are in $\Pi_{2}^{0}$ and not in $\Pi_{1}^{0}$. Turing acknowledged that he had no proof for believing the conjecture to be true. Feferman proved, on the contrary, that the class of $\Pi_{2}^{0}$ statements is incomplete in the sense of Gödel, and some of these statements are true but not provable in ordinal logics. In sum, Gödel’s incompleteness theorems remain true, and there is no way around incompleteness entirely, not even in a relatively constrained situation that seems nice.

There are many theorems that, when looked at in a particular logical system, can be stated and are true but are not provable. One theorem like this is Kruskal’s tree theorem: mathematicians can prove it, and in principle artificial computers cannot. Some context (without full detail) may be helpful. A tree is a graph without any loops, and Kruskal’s theorem is, ironically, an important tool in computer science (Gallier, 1991). It says that if we take the set of all finite trees and if we label each vertex in each tree from a certain kind of label set (called well-quasi-ordered, whose details are not necessary here), then those labeled trees form a set that is again in that same kind of label set.

This relatively simple-sounding theorem is possible to state in any logic system relevant here, but it is sort of surprisingly hard to prove: In fact, it is impossible to prove Kruskal’s theorem within simple logic systems such as second-order arithmetic (SOA), which our artificial computers use. By contrast, it is possible to prove Kruskal’s theorem within more sophisticated logical systems such as set theory (e.g., ZFC or ZF, short for Zermelo-Frenkel set theory with or without the axiom of Choice). Mathematicians can prove Kruskal’s theorem and the fact that this theorem is not provable within SOA, e.g., as an example of Gödel’s incompleteness theorem (Gallier, 1991).

Because a computing device cannot construct this proof in the current computing paradigm, this means that artificial computers arguably cannot understand the proof of Kruskal’s tree theorem. As an aside, they may understand other things. We use the word “understand” here in the linguistic sense of matching symbols or operations to objects or actions in the outside world, and consider the abstract objects of mathematics to be part of the world outside of artificial computers. It is true, however, that a computing device can check the validity of a proof in set theory, because there are finitely many axioms; that is fundamentally different from constructing the proof in the first place.

### 4.2 Mathematicians and other humans can do many things that are not computable

Mathematicians have a larger set of abilities beyond just proving theorems that are unprovable in artificial computers’ logic of SOA. In general, we can get outside of logical systems to define new logical systems. We can also imagine infinite things and to work effectively with the concept of infinity. Artificial computers cannot do these things; they malfunction because of infinite loops, a problem that humans do not have. And we can do basic reasoning that ANNs struggle with. For instance, GPT-4 and GPT-4V (a fancier, multimodal version) cannot reason abstractly like humans can, even with correct examples to help them (Mitchell et al., 2024).

For present purposes, it is enough that, due to incompleteness theorems, there is at least one human mathematical activity that artificial computers cannot do—at least within the current computing paradigm. Thus, no artificial computer can simulate all the mathematical activities of a human computer (using here the Turing sense of “simulate,” i.e., one computer fully duplicating the activities of another computer). This shows that there is a significant gap between humans and current devices at the macroscopic level.

## 5 Microscopic: gaps between biology and computer chips with cues for whole brain emulation

There are gaps between functions of biological computing and of electronic computing on the microscopic scale of molecules and transistors. Some of these microscopic gaps may help explain the macroscopic gaps that exist between humans and non-human computers.

### 5.1 A neuron is mesoscopic and more sophisticated than a logic gate, which is microscopic

For a long time, neurons have been compared to transistors or logic gates on microchips (McCulloch and Pitts, 1943). In current computing technologies, however, logic gates are made from combinations of modern types of transistors called MOSFETs (metal–oxide–semiconductor field-effect transistor). And transistors are a decent analogy for certain ion channels in neurons, rather than whole neurons.

Specifically, the biological ion channels that are most like transistors are voltage-gated ion channels, small pores in a cell membrane that can open or close according to the voltage sensed, to allow the flow of ions such as sodium through the pore, comparable to the way that a MOSFET works (Cheng and Hu, 1999). One main contrast with ion channels is that transistors stay where they are affixed to a chip: the only moving parts of transistors are the flow of air and electrons. (Air ideally carries heat away from the chips with the help of good ventilation, because otherwise the CPU eventually gets fried, as happened to me once on my way to a job interview.) By contrast, biology’s ion channels can move around in the membranes of cells (including, but not limited to, neurons). In particular, ion channels can cluster together in useful ways (Dixon et al., 2022; Pfeiffer et al., 2020).

A typical neuron has many thousands of ion channels, including ion channels governed by neurotransmitters instead of voltage, so the neuron’s scale is closer to a whole microchip rather than a single transistor or logic gate. This is true in function as well as structure, because efforts have been made to use artificial neural networks to model the behavior of one neuron, and the number of nodes required for the model ANN is around several hundred (Beniaguev et al., 2021). And even that simplifies the full behavior of a neuron.

One of these other kinds of ion channels is broadly considered to be like an AND logic gate: the NMDA receptor, because both NMDA (N-Methyl-D-Aspartate, an amino acid similar to glutamate) and glycine must bind for its ion channel to open (Hollmann and Heinemann, 1994). It is a bit more accurate to say that the NMDA receptor is more like a half-adder. But even that is a reduction of what the NMDA receptor actually does. In reality, serine can bind instead of glycine; zinc and magnesium can block the receptor briefly between action potentials; and a number of drugs are psychoactive because they can block the receptor, such as alcohol, dextromethorphan (a cough medicine known to cause hallucinations), and PCP (phencyclidine). As a result of these nuances, the NMDA receptor works on multiple time-scales and is more complex than a standard logic gate (Gribkova and Gillette, 2021).

Another important part of a neuron is its dendrites, or receptive branches, which are not at all the passive transmitters of information that they have traditionally been described as. At the tips of dendrites are post-synaptic densities (PSDs), which are proteins assembled into scaffolds that attach to the inside of a set of receptors (Wilkinson and Coba, 2020). These PSDs have many possible configurations of many kinds of proteins, and among other things, some of these PSD proteins send signals to the center of the neuron, for example, by second messengers such as cAMP.

This makes it clear that dendrites are not passive and simply receive neuronal signals in relay to the neuron center (a.k.a. soma); rather, dendrites are active and process incoming signals to send a variety of signals onward, such as waves of ions (including some action potentials backward up the dendritic tree) and waves of small molecules (e.g., cAMP). The neuron soma, in turn, receives and sends signals onward, such as action potentials, neurotransmitter packets along microtubules (including to the axon end), and newly made proteins from changes in gene expression.

A number of aspects of cognition rely on gene transcription, and more generally, on gene expression. One example is long-term potentiation (LTP), where repeated stimuli cause transcription and translation which result in long-term (more than a day) synaptic changes (Lee et al., 2008). Mammals need these changes in order to consolidate our learning. Other examples are from studies with knockout or knockdown organisms to repress gene expression or transcription. For instance, two layers of gene transcription matter in a double knockdown experiment in mice (Li et al., 2019), where one gene is epigenetically suppressed and a second gene is suppressed another way; when the second gene is un-suppressed, its corresponding protein repairs the epigenetic DNA damage, so that the first gene can be transcribed and expressed and the mouse can consolidate its memory. These genes are neuronal immediate-early genes (IEGs) and are studied in the fields of neurogenetics, and cognitive genomics (Minatohara et al., 2016). Knockout studies in human stem cells show that coordinated transcription and expression of DLG2 and related genes are crucial in cortex development: changes to this coordination affect PSD proteins, and some of these gene expression patterns have a higher risk of schizophrenia (Sanders et al., 2022). Rat studies on DLG2 genes reveal some mechanisms, including impaired LTP (Griesius et al., 2022).

All of these neuronal activities imply that a neuron is significantly more sophisticated than a logic gate. Thus, we must look at scales smaller than neurons to compare and contrast the computing abilities of microscopic biological structures and electronic microchips. Specifically in the following, we will look at biological molecules such as ion channels, other proteins, nucleic acids, and smaller molecules used as signals—to analyze the relative computing abilities comparing and contrasting them with transistors and logic gates.

### 5.2 Cells use more particles in more ways than electronics can

Many more kinds of ions flow in biochemistry than in electronic chips. The term “electronics” comes from electron flow in these devices. (The name “Proton Mail,” sadly, does not correspond to proton flow). Biology makes crucial use of electron flow, notably in the Electron Transport Chain (ETC) of mitochondria for making cell energy in the form of adenosine triphosphate (ATP). There are other ways that electron flow plays an important part in biology, for example, in photosynthesis, and more generally, redox reactions (including, e.g., UV damage to our DNA). And electrons may play other important roles in biology, such as moving along microtubules, which are large protein complexes that may support significant topological electronic states (Subramanyan et al., 2023).

Biochemistry makes use of many other ions and charged particles and their flows around cells, especially across cell membranes. These include protons (H^+^), metal ions (e.g., Li^+^, Na^+^, K^+^, Mg^2+^, Ca^2+^, Zn^2+^, and, in the astronomers’ sense of metallicity, Cl^–^), and molecular ions (e.g., nicotinamide adenine dinucleotide, NAD^+^, and its relatives; phosphate, the P in ATP, or adenosine triphosphate, which is a store of energy). These metal ions are the ones that flow through ion channels in neuron membranes, as spikes.

Gases also flow around in biochemistry, and in more sophisticated ways than just cooling for electronics. Famously, oxygen and carbon dioxide flow in their cycles, and more interestingly, nitric oxide diffuses from neurons to dilate nearby blood vessels. Other fluids flow around too, notably water and the small molecules that can diffuse easily through water and cytoplasm, such as ATP and its relative cAMP. Notably, the “A” in ATP and cAMP are the same molecule, adenine, and the same molecule that appears among the genetic nucleotides A, C, G, and T.

With all of these ions and small molecules flowing, biochemistry has much more flexibility and function than only electrons flowing in the hardware of electronics. For example, many biological ion channels are selective, allowing only certain kinds of ions to pass. And ion channels can cluster together without quantum tunneling interfering with their workings, because protons and metal ions can tunnel only a tiny fraction of the width of the molecules.

### 5.3 Mitochondria are important in brains and use electrons better than electronics can

Mitochondria produce ATP, famously for metabolism, and they also do a lot more. For instance, mitochondria play main roles in regulating reactive oxygen species, which act as second messengers in signaling pathways (Bernardo et al., 2016); regulating neurotransmission by buffering calcium (a useful but corrosive ion that needs to be tightly controlled, Billups and Forsythe, 2002); regulating synaptic plasticity via neurotransmission control (Mattson et al., 2008); regulating hormone production, which has many effects (Spinelli and Haigis, 2018); and regulating cell death, which we usually want for cancer cells and not for healthy brain cells (Morella et al., 2022).

Metabolism plays an important role in cognition, research has shown. For example, in mice mitochondria, boosting the expression of two genes, glyoxalase 1 and glutathione reductase 1, results in increased anxiety. And interfering with the expression of the first one, glyoxalase 1, reduces anxiety behaviors in the mice (Hovatta et al., 2005, and follow-on work). Emotions such as fear and anxiety are important for attention and decision-making in cognition (LeDoux et al., 2009), probably through links to ancient states of aversion to noxious stimuli and attraction to positive stimuli.

Also, in the metabolic process of making ATP, biology arguably makes better use of electron flow than does a computer chip. The biological Electron Transport Chain (ETC) takes advantage of a key quantum aspect of electrons, which is their ability to tunnel from one protein complex to another, across distances of around 10 nanometers. In fact, a source of cellular dysfunction is due to the protein complexes of the ETC changing shape (e.g., by aging or oxidation, Korovila et al., 2017) and spreading apart, which makes it harder for electrons to tunnel and results in less energy for the cell.

By contrast, quantum tunneling is a problem, not a benefit, for packing transistors onto a computer chip, because if the transistors are too small or too close together, then there are random leaks that ruin how reliable the chip is. There is a limit on the size of a transistor (called gate length) is around 10 nm, so gate lengths in chips have been above 14 nm starting in the mid-2010s. There is also a limit on the distance between transistors (called gate pitch), possibly similar to the gate length limit, although in practice, gate pitches have been around 50 nm in the 2020s. Notably, marketing terms such as “the 7 nm process” have departed from any relevant sizes of or distances between actual transistors.

Comparing the quantum possibilities in the ETC and gate pitch may seem unfair, since it is not electrons that are used in neurons for spikes, but rather larger ions flowing through channels. This is true: and biochemistry makes use of many charged particles and of their flows across cell membranes. These ion channels still have an edge over transistors: ion channels may be closer together without the interference of quantum tunneling, because the ions are much larger than electrons.

As for the energy sources of artificial computers, it is notable that they use electric currents for both power and for signal or information processing. This dual-purpose nature of electricity is similar to biology’s use of the same ions in both power production and information processing. There is a limit, however, on the amount that an electronic computer can throttle up or down its energy, and overheating is a serious risk. By contrast, an organism can adapt to many levels of energy, and can actively change the amount of energy available to itself or to specific areas of its brain. Overheating is also a risk in biology and can cause changes in cognition, but controlled overheating also serves the crucial function of fighting infections.

In light of the endosymbiotic hypothesis, that mitochondria were early bacteria that early prokaryotes incorporated as symbionts, proposed by Lynn Margulis in 1967, it may be especially interesting as electronics incorporate power production components (more than power-storage components like batteries) as part of their inherent functioning. On a larger scale: what if data centers swallowed up entire power plants, and made more clever use of the power than both supplying power to the electronics and also supplying power for cooling the resulting overheated electronics?

### 5.4 Estimating the brain’s power implies that we may emulate it around 2040

There are several possible meanings of “emulating” a brain, ranging from so-called “weak AI” which would model a whole brain, up to AGI or “strong AI” which would replicate a brain and all its main functions. For all of these meanings, a good starting point for estimating when we may be able to emulate a brain in our future technology is through counting the operations per second (OPS) that biological molecules can do and then scaling up by the number of such molecules in a brain. Such a calculation may go as follows.

We will get a minimum estimate for how many fundamental computing units are in a human brain, by counting up brain cells and their ribosomes, proteins, and lipids. At the same time, we will estimate how many operations per second each of these molecules can do. To start, a human brain has about 10^11^ cells, including around 86 billion neurons (Herculano-Houzel, 2012) and around 72 billion glia, which are as important as neurons in our brains’ functions (von Bartheld et al., 2016).

Each brain cell has about 10^6^–10^7^ ribosomes (Dastidar and Nair, 2022) and about 10^7^ proteins. This latter is consistent with each post-synaptic density having about 1,000 proteins (Sheng and Hoogenraad, 2007), and each neuron having synapse numbers ranging across neuron types from about 2,000 for retinal ganglion cells to over 100,000 for Purkinje cells (Demb et al., 2004; Harvey and Napper, 1991). Thus there are a total of at least about 10^7^ computing units per brain cell in the form of proteins or ribosomes. Next we estimate proteins’ number of operations per second.

Each protein in a brain cell may be able to do about 100 operations per second (OPS). In general, proteins can change their shapes or conformations on timescales of 0.1 ms (which is 10^–7^ s) to seconds. There are many important molecules in brain cell functions, and proteins are only one type. Lipids are another, nucleotides another, and ribosomes yet another. All of these can be thought of as fundamental units of biological computing, and we will focus on proteins and ribosomes as fundamental units for this calculation.

Ion channels operate sort of like switches on the millisecond (ms) scale, with a single switch happening in about 20 μs (2% of a millisecond) for certain acetylcholine channels (Unwin, 1995). These protein operations are basically binary, opening or closing. But changes in channel tendencies, such as desensitization, allow more complex activities in these proteins, which happen on slower timescales (but still fast by our standards), around 100 ms in neurons (Yu et al., 2009). Some ion channels are selective for one kind of ion; others allow several kinds through, which can be thought of as non-binary operations.

Other receptors (such as G-protein coupled receptors, or GPCRs) are slower, operating on a scale ranging from 0.1 s to hours, sometimes faster by acting on nearby ion channels, and they can desensitize on the order of seconds to minutes or longer (Huang and Tesmer, 2011; Kobilka, 1992). These GPC receptors have more than just two states, for example, causing signals by cAMP or lipids. GPCRs are targets of about half of all pharmaceuticals—modulating GPCRs can cause many therapeutic effects. Other proteins have multiple states, too, such as CaMKII (Calcium/calmodulin-dependent protein kinase II), called molecular memory and associated with long-term potentiation in synapses, which has about a dozen states (Luciæ et al., 2008).

Averaging over all these proteins, with their different numbers of states and different timescales of operations, is what leads to proteins’ 100 OPS estimate: Ion channels may do about 2 operations per 20 microseconds, which amounts to 10^5^ OPS. GPCRs and others may do about 4 operations per 0.1 s which amounts to 40 OPS. The geometric mean of these figures, 10^5^ and 10, is around 100 operations per second.

Ribosomes’ speed of operation is well-studied: they translate each of 64 codons to each of 20 amino acids at a rate of about 50 ms per codon, and they also help protein-folding which may happen as fast as microseconds (Inafuku et al., 2023). If we view selecting the correct amino acid as one operation with 20 different possible states, the ribosome is capable of around 20 translating operations per 0.05 s = 20 * 20 operations per second = 400 OPS, and additional folding operations per second. This number of operations is similar to proteins, on the order of 100 OPS.

Ergo, with about 10^7^ ribosomes and proteins per brain cell, we will estimate their OPS to be around 10^9^ per cell. With some 10^11^ human brain cells, there are about 10^20^ operations per second in the human brain. And that is not counting operations done by things other than ribosomes and proteins.

May other molecules contribute significantly to the brain’s operation too? Lipids are also important computing units in each cell, and there are thousands of types of lipids, including for our purposes, signal lipids and regulatory lipids. Lipids have been historically less studied and less appreciated than proteins; biologists thought lipids were inert until the work of Mabel Hokin and Lewis Hokin with radioactive phosphorus (Hokin and Hokin, 1953). Some lipids in a cell membrane may indeed be “only” structural, but many others that appear structural also turn out to play important regulatory roles. For example, two ways that GPCRs are regulated is through internalization and endocytosis, where part of the structural membrane around some receptors pinches off temporarily or permanently inside the cell (Yu et al., 2009). This causes the cell to be desensitized to some of the neurotransmitters that the receptors would have bound, an important part of neuronal plasticity (Tsao and von Zastrow, 2000).

The typical surface area of neurons (and astrocytes) may be around 2,000 mm^2^, or 2 * 10^9^ square microns (Dai et al., 1998). The number of lipids per square micron of membrane is around 5 million (van Meer et al., 2008; Vance, 2015; Alberts et al., 2022). Thus, the total number of lipids in the membrane of a neuron or astrocyte is around 5 * (10^6^) * 2 * (10^9^) = 10^16^. And there may be as many again in all the other membranes of the cell.

Granted, only a small proportion of lipids do computing, but a healthy number of them are at least the computational equivalent of switches. Of the thousands of types of lipids, dozens are known to be signals or regulators (Hannun and Obeid, 2008; Davletov and Montecucco, 2010), so if 1% of lipids do computing like this, this amounts to 10^14^ lipid computing units per cell. As for how fast each one operates, one operation per second is a decent minimum, as follows.

Let’s consider the lipid IP_3_, which is a small molecule about 1 nanometer (nm) across, which can diffuse, or move, about 20 microns during its lifetime of a few seconds (Allbritton et al., 1992). For comparison, a protein that is 5 nm across can diffuse, or move, about 3 microns in 0.1 s. The target receptors of IP_3_ are on endoplasmic reticulum (ER), which is distributed in the cell, including in the dendrites, dendritic branchlets, and dendritic spines, where IP_3_ is likely to be released. A dendritic spine is about 1 micron around, and a dendritic branchlet is about 10 microns long, so each IP_3_ molecule generated in a dendritic spine can move far enough to reach its signaling targets, including some 20 microns away in the dendritic branches beyond its own branchlet.

As a result, we could estimate the total number of operations per second done by lipids to be 10^14^ operations per second per cell, and 10^25^ OPS per human brain. This may seem counterintuitive by comparison to proteins’ total computing power of around 10^20^ OPS, but it makes more sense in light of the fact that lipids are much smaller and much more abundant than proteins. So even if lipids are not as fast or as computationally active as a typical protein, they still have a great deal of combined computing power.

Many of these computing operations are not in our conscious awareness, because our brains do a lot of background work, including while we sleep or think about other things, work that eventually benefits our conscious brain function. And all of these estimates are approximate and provisional: they can be updated as we learn more about the molecular biology of brain cells, whether neurons or otherwise. Exosomes of RNA, for example, are a newly seen way that neurons communicate (Taylor and Nikolaou, 2024).

Shifting now to compare with supercomputing technologies, zettascale computing will be capable of about 10^21^ operations per second, anticipated to happen around 2030. One may expect that zettascale computing will be enough to emulate a whole human brain, but given than the brain operations calculation was an underestimate, this expectation may be too optimistic. It is also believed that zettascale computing will be able to model supernovae and black holes more effectively and will be able to forecast 2 weeks of weather globally (DeBenedictis, 2005), so if we believe that the brain is more complex than weather and astrophysical phenomena (and I do), then we should be skeptical that zettascale computing will be enough for brain emulation. Brain emulation may well require more, namely yottascale computing, or 10^24^ OPS, around 2040.

## 6 Human ingenuity probably comes in part from our multiscale neurobiology

Our brains do many things on many space- and time-scales, and they are broadly important for human ingenuity; yet, historically, certain scales have been championed (mesoscale/microscale neurons) and others have been overlooked (nanoscale molecules and proteins, megascale bodies and communities). To be fair, nanoscale neurotransmitters have been considered important, but they have often been viewed as only bridging neurons’ spikes. They are, however, more than bridges between spikes, because neurons release many neurotransmitters from their dendrites or somas (Ludwig et al., 2016; Welle et al., 2018), affecting nearby cells without necessarily involving spikes.

We also know that subneuronal activities are important in their own right, as in examples of proteins or lipids discussed above. Specifically, microscopic molecules like CaMKII play a role in memory that is different from the macroscopic hippocampus and its mesoscopic neurons. (Of course, hippocampus neurons have CaMKII proteins, which may be especially important to their functions). These subneuronal structures and activities sometimes have little correlation with the more easily observed spiking activity of neurons, although new neurochemistry methods have emerged to probe nanoscale activity (Chen et al., 2020). An entire category of neurons, called silent or dark neurons, have begun to be appreciated for their activity, which is surely crucial for brain function even if it is not as spectacular as firing (Shoham et al., 2006; Wohrer et al., 2013).

### 6.1 Biology’s interacting across scales boosts ingenuity and efficiency

Interactions across scales are important because they can amplify ingenuity and can efficiently process things outside of conscious awareness. The macro-scale brain can affect micro- and nano-scale structures in a process called top-down causation (Walker et al., 2016). I propose an example of top-down causation: the tip-of-the-tongue phenomenon, when we know a word we want to say but cannot access it (at least not fully), then we do something else, and later the word appears easily when we try to access it again. Between trying and succeeding there is time for processes, below conscious awareness and likely microscopic or mesoscopic, that began due to the initial macroscopic effort of the person.

This ability, our brains’ doing intensive memory-recovering while we are consciously doing other things, has only partial parallels in artificial computers, such as recovering corrupted memory; but in contrast to the biological processes, recovering corrupted memory takes a great deal of conscious effort by the user and sustained energy by the device, and it is certainly not something that the device can do easily and on its own. Of course, corrupted device memory is not the same as the tip-of-the-tongue phenomenon; there may be other parallels.

Another way that biology can amplify ingenuity is through interactions on megascales, i.e., scales larger than brains. One example of a megascale interaction is an organism using its brain, the rest of its body, and some parts of its environment, all to solve challenging problems that the brain alone cannot solve (at least not easily). Cooking, writing, gardening, and using calculating tools are instances of this. Among non-human animals, salamanders and birds use their magnetic senses for migration, interactions on a planetary scale.

Another example of a megascale interaction is when many organisms (often of the same species) work together to overcome a challenge that one organism alone cannot. Hunting in packs, social learning, and slime molds moving are instances. Whales provide a vivid example of social learning, with their success in evading whaling ships in the 1800s (Whitehead et al., 2021). Within a few years after the first whaling ships came to a region, the whaling success rates dropped by about 60%, a drop that cannot be explained well by whaling harpooners’ skill or by the vulnerable whales’ deaths. Instead, the best explanation is that whales learned how to avoid whaling ships and taught each other that valuable skill. This kind of learning is an example of animal culture and had the particular result of protecting vulnerable individuals. Whale communication also helps to improve migration, including communication over long distances (Dodson et al., 2024). Organisms also process a wider variety of inputs and outputs than artificial computers do, even wider than the multi-modal “Frontier” models do, and organisms integrate this variety in organic ways that devices cannot.

### 6.2 Lessons for making more ingenious and efficient computing devices

We would do well to be inspired by these examples from nature of animals and other organisms that are ingenious and efficient by combining computing abilities with actions of their bodies and interactions with their peers and environments. There are several efforts along these lines that are worth highlighting and considering how other systems could be constructed to hew more closely to biological computing, in order to achieve the goal of synthesizing AGI with neurobiology. It would be fruitful to imitate biological systems that have simple parts but complex collective behavior, such as slime molds, swarms of insects, and schools of fish. Groundbreaking work like this includes models of simple-but-complex systems, including active matter, multi-agent systems, and swarm robotics (Werfel et al., 2014; McCreery et al., 2022; Ko et al., 2023).

These models are part of the larger field of amorphous computing, whose goal is to “draw from biology to help create and entirely new branch of computer science and engineering” to orchestrate many simple and local devices for solving problems (Abelson and Forbes, 2000). A classic inspiration in this field is slime molds, which have networks of tubes for sending molecules as signals, to move in concert using oscillatory space-time waves. This is similar to (but not a precursor of) nervous systems that also have oscillations and space-time waves (Boussard et al., 2021). Slime molds can also learn through habituation when exposed to caffeine, an analog of the neurotransmitter adenosine (Boisseau et al., 2016). Similarly, there are tiny nerve-less organisms that use neurotransmitters to coordinate their movements, an example of robust collective behavior (Jin M. et al., 2024).

To be sure, artificial neural networks are collections of many simple and local devices (neurones or nodes) for solving problems. But the entire ANN is considered the agent; whereas in amorphous computing, each local device is an agent that can move around to interact with the environment and with the other devices, a paradigm that is quite different from the fixed locations of ANN nodes and their limited interactions with each other (e.g., with two other layers of nodes) and with the environment (through electricity, incidental heat production, and airflow for cooling). The most significant interactions of ANNs are with people: the people who program them, and give them feedback for reinforcement.

Making artificial neural networks bigger and deeper will not evidently achieve our goal of synthesizing AGI with neurobiology, because, for instance, such AI models have a problem adapting to new information: they tend to do badly on new tasks apparently because of earlier training on previous tasks, even if or especially if they did well previously (Dohare et al., 2024; Sun et al., 2024). This is why tech companies tend to discard old AI models and train new ones from scratch for a new purpose, and this may also be why Google search and Chat-GPT seem to get worse (Bevendorff et al., 2024; Chen et al., 2024).

Similarly, expanding AI models another way by adding actions (or putting ANNs into robots) may not achieve this goal, although this kind of “embodied AI” is a current effort, and it is at times disastrous, as in the case of autonomous vehicles (e.g., Siddiqui and Merrill, 2023).

On the other hand, small-scale and narrowly targeted robots show promise in medicine. Remote-controlled nanobots may be able to deliver localized treatments that would otherwise be toxic if taken orally in sufficient quantities to treat the target tissue (Landers et al., 2025; Han et al., 2024). Notably, these kinds of nanobots still require human guidance, in the form of magnetic control. Perhaps in the future, they will be able to be more autonomous, for instance, by following chemical gradients (Zheng et al., 2023). We need to thoughtfully integrate inputs with outputs and actions with computations, considering what we need to do and how to do that well.

## 7 Discussion: we need a computing paradigm shift to achieve biologically loyal AI

The current efforts to achieve the stated goals of AGI are largely *ad hoc*, without the support of a coherent theory. AI has many apparent successes and apparent guardrails; they dissolve when pushed cleverly but realistically (Jin H. et al., 2024). There is a good argument for keeping AI systems small and narrow, each tailored to a specific goal, which would likely be more robust and have fewer pitfalls (Bender et al., 2021). If, however, we want good examples for making functional broad AI/AGI systems, we could do well to study two kinds of ingenious systems: naturally occurring biological systems that are robust and adapted to their environments; and hybrid biological/electronic systems developed over time that are also robust and useful for their purposes.

### 7.1 Inspirations for a new synthesis: simple biological systems and hybrid systems

If we want to understand the gap between AI and biological ingenuity, then it is good to study systems and models accounting for embodied cognition, because every natural nervous system exists in a body and environment, and because every meaningful model of a brain must treat it as non-isolated. At a minimum, brains receive many kinds of inputs and outputs, many more kinds than are mimicked in artificial computers. Brains receive inputs from the senses, and in addition, inputs such as nutrients; brains send outputs such as language creation, and in addition, outputs such as hormones that may influence other biological systems.

To understand something, mathematical physicists often choose the simplest non-trivial example, called a toy model and used to thoroughly understand a concept or mechanism, with only the necessary complications. Our brains are far from toy models that we can understand well enough for building artificial equivalents; better for this purpose is the lobster stomatogastric ganglion (Calabrese and Marder, 2025).

The stomatogastric ganglion (STG) has about 30 neurons, and it has many features that give us insight into our brains and are easier to imitate in technology than our brains. Fully imitating the ganglion in silicon chips is not possible, however, because one of its main functions is to physically “chew” the animal’s food to help extract nutrients (nutrients that can then travel through the blood and reach the ganglion itself, possibly changing its biochemical and electrical properties). Silicon chips are not able to physically move this way, nor to contribute to the changing of their electrical properties. Also unlike electronic devices, the STG is both flexible and robust (Prinz et al., 2004; Grashow et al., 2009). It is flexible, because each animal develops and adapts its neural circuit properties to do digestive tasks in its own way. And it is robust, because each animal’s STG can maintain homeostasis, keeping its functions steady even in the face of changes.

The heart and the Venus flytrap also manage to be robust and flexible biological computers (Kirkpatrick, 2022). Other examples of simple-but-complex biological systems are slime molds, swarms of insects, and schools of fish. It can be a surprising challenge to imitate in technology some of these apparently simple biological computing systems, a challenge which can be seen in the steady progress of medical pacemakers. A key benefit from emulating these simpler biological systems instead of the brain—in addition to their being more tractable—is that they force artificial materials to combine computing and moving, which life does well and has done well since the beginning.

It is worth noting that the academic fields of AI and artificial life (A-Life) were the same academic field until they diverged for political reasons around 1960, into symbolic computing (mind) and cybernetics (life and networks), the networks part of which plunged into a winter caused by symbolists in 1969 (Boden, 2016). Networks came back into fashion in the mid-1980s, around the time that the subfield A-Life got its name. The divide between AI and A-Life is largely artificial, because all known natural “intelligence” is embodied in life forms, and thus in some sense, AI and A-Life and AGI should all be the same sort of thing, and all embodied.

Other sources of inspiration are hybrid systems, which combine biological and electronic features. These would also help us formulate and test a paradigm-shifting theory that synthesizes the theoretical foundations of neurobiology and AGI. Emulating a hybrid system in artificial materials would be a challenge, but part of the emulation is easy because the engineered part of the hybrid system is already an exact model of itself. Examples of hybrid bio-electronic systems include: surgeons using robots, data workers assisting LLMs through RLHF algorithms, disabled people using sophisticated assistive devices such as BrainGate2 (Vogel et al., 2014), and more generally, Human-Computer Interaction (HCI). Other bio-electronic hybrid systems include some experimental setups in neuroscience and some types of hacking that rely on non-computing actions invented by people.

Studying these hybrid systems as models for a new synthesis will be different from research done to advance the hybrid systems themselves, but there will almost surely be fruitful cross-pollination between the two types of research. Watching the biological and the artificial parts of the system interact should help us understand and emulate the biological part better. An example of a hybrid system is the use of humans to train LLMs in the paradigm of Reinforcement Learning with Human Feedback (RLHF).

There are also scientific approaches and engineering technologies that could inspire a unified theory, including amorphous computing, “smart” materials, soft condensed matter physics, and physics-based hacking such as Rowhammer. In addition to providing examples for a theoretical synthesis, these technologies may help form a practical synthesis of neurobiology and AGI.

On the practical side, there are candidates for a technological synthesis of AGI and neurobiology. Neuromorphic computing, when it uses current computing frameworks, will probably not suffice to accomplish AGI. Neuronal mechanisms in biology are much more nuanced than computing technologies, so truly neuromorphic computing would require materials that are more multivalent than electrons and silicon. There is an effort to put neurons on chips, which is interesting for basic science, but for AGI would still need meaningful embodiment (Amirifar et al., 2022; Brofiga and Massobrio, 2022; Buentello et al., 2024). Synthetic biology is a fruitful counterpart to pursue as a complement to the computer-engineering approaches (e.g., Robertson et al., 2025).

### 7.2 Hypothesis for a new synthesis of AGI and neurobiology in theory and in practice

There have been calls for a unified theory of biological computation and artifact computation (Anderson and Piccinini, 2024, section 10.4). Combining AGI and neuroscience into a synthesized theory may not be as simple as patching physical actions onto computing systems. Theories can be patchwork and useful at the same time, however: consider the successful but quilted theories of partial differential equations or large deviations theory in mathematics. If we were to stitch two theories together for Neurobiology and AGI, ones that would make sense are computability and control theory. Control theory is about action and other physical effects, and it does a good job of simulating homeostasis. It is an open hypothesis, however, whether such a resulting patchwork theory would achieve what we would want, because there’s no obvious source of creativity or imagination in either of the two theories (at least not beyond pure randomness), and thus no evident way to achieve something like biological ingenuity. In any case, a theory that synthesizes foundations for neuroscience and AGI must cover both electronic computing and biological computing, and must ultimately describe all the important things that mathematicians do—more than just pen-and-paper calculations—especially the things that involve action and imagination.

The present proposal is: For humans, cognition is fundamentally entangled with motion, including action within and interaction with our environments (physical and social). This entanglement holds at all scales, including micro and macro and in between. And there are causal links between the entanglements at different scales, links that go in all directions. This theory could eventually be a statistical mechanics of the brain, with complexities beyond what we usually find in the statistical mechanics of gases or ultracold atoms in physics. Some of these additional complexities already exist at the small scales (quantum chemistry and protein folding are notoriously hard to explain); some of them may emerge from collective actions at smaller scales; others may not (human choice being a famous example).

At the microscopic level of logic gates or conditional branching algorithms, boosting Turing machines to include action should help bridge the theory gap between artificial and human computers (Kirkpatrick, 2022). There is some evidence of this hypothesis from microscropic interactions of simple organisms in a “social” environment, including the fact that some neurotransmitters and their analogs are used by nerve-less organisms to communicate, coordinate movement, and learn in the right conditions (Jin M. et al., 2024; Boisseau et al., 2016). Our neurons hold more evidence, because the motion of our mitochondria along microtubules is crucial for cell functioning, including learning through synaptic plasticity (Hollenbeck and Saxton, 2005; Mattson et al., 2008). At this level, we should appreciate that sub-neuronal computation and action are important, and we should also appreciate neurons that don’t seem to be doing much but almost surely are actually doing a lot (called “dark neurons” or “silent neurons”).

At the macroscopic level of the individual, there is also evidence that cognition is entangled with motion: Exercise is linked with brains working well (e.g., Bernardo et al., 2016). This is also a causal link between the microscopic and macroscopic levels through, e.g., mitochondria. There is more evidence of cross-level linking, such as variants in a neuronal ion channel from the ATP1A3 gene and rapid-onset Parkinsonism disorder, which involves changes to both motion and cognition (e.g., Brashear et al., 2012).

Larger than the individual level, social interactions and environment interactions are similarly important for cognition, and bodily motion is both a cause and effect of cognition at this level. Persuasive arguments have been made that specific aspects of situated cognition should be appreciated more, specifically olfactory and biosocial aspects (Jacobs, 2023; Lewis, 2020). In addition to lifting up these perhaps under-appreciated standpoints on 4E cognition, I propose that they can be integrated into the same hypothesis, one that integrates motion with cognition. Olfaction at the microscopic level involves molecules moving to fit hand-in-glove (the lock-in-key metaphor is too rigid), and at the macroscopic level involves major histocompatibility complexes influencing interactions between individuals. And between levels, olfactory stimulation often triggers memories and supports social learning that requires motion, such as motion toward food locations (Sullivan et al., 2015).

At all levels, more subfields of mathematics will be required than those subfields that are amenable to computational representation, e.g., partial differential equations, dynamical systems, geometry, and topology. To an extent, some of these subfields are already being used in neuroscience models (e.g., Curto and Sanderson, 2025). Other considerations are that computational activity is not identical with mathematical activity, and that non-computational activity is as important as computational activity. Integrating computational and non-computational processes, e.g., of the brain, will result in more than the sum of the parts. This should be true for technology as well, and the integration is a challenge.

A consequence of this proposed hypothesis is technological: Any successful imitation of our cognition must involve many kinds of moving parts and many kinds of motion, emphasizing robotics over LLMs, but more advanced than classical robots and more integrated than existing “embodied AI.” The Mars landers and rovers are excellent starting points. Another excellent starting point is the use of bio-electronic hybrid systems in a clever experiment on the lobster stomatogastric ganglion (STG), with two-cell hybrid circuits, one cell a neuron in the animal, and one a simulated “neuron” using dynamic clamping (Grashow et al., 2009, 2010). This hybrid system confirmed the prediction that the lobster STG is robust, able to compensate for changes from the simulated cells to achieve mesoscopic and macroscopic behaviors that were as useful as before the addition of the dynamic clamps. This kind of work should be supported more.

We have a long way to go from engineered cells and industrial robots to engineered minds. To quote Evelyn Fox Keller, “The bottom line is that with every passing achievement—in biological computing and computational biology—the gap between computers and organisms becomes both ever narrower and more elusive” (Keller, 2002). After all, the only computers that can do the mathematical reasoning around incompleteness and set theory are biological computers. So far.

## Data Availability

Publicly available datasets were analyzed in this study. This data can be found at: https://kirkpatrick.web.illinois.edu/; https://pubmed.ncbi.nlm.nih.gov/; https://arxiv.org/.
